# Extracellular vesicles from human bone marrow mesenchymal stem cells repair organ damage caused by cadmium poisoning in a medaka model

**DOI:** 10.14814/phy2.14172

**Published:** 2019-07-19

**Authors:** Tomomi Matsukura, Chisako Inaba, Esther A. Weygant, Daiki Kitamura, Ralf Janknecht, Hiroyuki Matsumoto, Deborah P. Hyink, Shosaku Kashiwada, Tomoko Obara

**Affiliations:** ^1^ Department of Cell Biology University of Oklahoma Health Sciences Center Oklahoma City Oklahoma; ^2^ Department of Life Sciences Toyo University Gunma Japan; ^3^ Department of Biochemistry and Molecular Biology University of Oklahoma Health Sciences Center Oklahoma City Oklahoma; ^4^ Clinical Proteomics and Gene Therapy Laboratory Kurume University Graduate School of Medicine Kurume Japan; ^5^ Department of Medicine Baylor College of Medicine Houston Texas

**Keywords:** Bone, cadmium, chronic kidney disease, extracellular vesicles, medaka, mesenchymal stem cells

## Abstract

Treatment modalities for kidney disease caused by long‐term exposure to heavy metals, such as cadmium (Cd), are limited. Often, chronic, long‐term environmental exposure to heavy metal is not recognized in the early stages; therefore, chelation therapy is not an effective option. Extracellular vesicles (EVs) derived from stem cells have been demonstrated to reduce disease pathology in both acute and chronic kidney disease models. To test the ability of EVs derived from human bone marrow mesenchymal stem cells (hBM‐MSCs) to treat Cd damage, we generated a Cd‐exposed medaka model. This model develops heavy metal‐induced cell damage in various organs and tissues, and shows decreased overall survival. Intravenous injection of highly purified EVs from hBM‐MSCs repaired the damage to apical and basolateral membranes and mitochondria of kidney proximal tubules, glomerular podocytes, bone deformation, and improved survival. Our system also serves as a model with which to study age‐ and sex‐dependent cell injuries of organs caused by various agents and diseases. The beneficial effects of EVs on the tissue repair process, as shown in our novel Cd‐exposed medaka model, may open new broad avenues for interventional strategies.

## Introduction

The heavy metal cadmium (Cd) has a half‐life of over 30 years in the adult human, meaning that even exposure to low concentrations can have lasting health consequences (Huff et al. 2007; Lane et al. 2015). Cd binds to metallothioneins and accumulates in the kidney, liver, and other organs, where the metal triggers cell toxicity. Damage is severe in the kidney, causing poor absorption of calcium, which is required for healthy bone maintenance (Sabolic et al. 2010; Orr and Bridges 2017). The precise age‐ and sex‐dependent mechanism that underlies the increased incidence of kidney injury for both sexes and various ages, and osteomalacia only in postmenopausal women upon chronic Cd exposure is vastly understudied, and no effective treatment is available (Aoshima 2016). Recent work with all‐male mouse and rat models has confirmed that exposure to Cd can mimic some, but not all, aspects of the Cd pathotoxic phenotype in humans (Prozialeck et al. 2007; Edwards and Prozialeck 2009; Prozialeck et al. 2009a; Prozialeck et al. 2009b; Yamanobe et al. 2015; Prozialeck et al. 2016).

Extracellular vesicles (EVs), a heterogeneous population of microparticles that are released by all living cells and carry various materials as cargo, play important roles in intercellular communication in normal physiology, disease pathology, and regeneration (Ferguson and Nguyen 2016). Materials found in the EVs of the animal kingdom comprise 1–5% of animal genes products (Liu and Clark 2012). One of the most attractive features of EVs is their ability to transfer the cargo to recipient cells and modify cellular phenotypes. Recent studies showed that human bone marrow mesenchymal stem cells (hBM‐MSC)‐derived EVs deliver complex biomolecules, such as microRNAs (miRNAs), mRNAs, noncoding RNAs (ncRNAs), proteins, and lipids that are beneficial for tissue regeneration, as shown in animal disease models (Bruno et al. 2009; Gatti et al 2011; Biancone et al 2012; Bruno et al. 2012; Quesenberry et al. 2015; Bruno et al. 2017). Notably, biologic materials carried by EVs are protected from enzymatic degradation in the physiological environment (van Niel et al. 2018). Published evidence indicates that hBM‐MSC‐EVs prepared using ultracentrifugation methods show some potential for kidney regeneration in mouse models of acute kidney injury (Bruno et al. 2009; Gatti et al. 2011; Biancone et al. 2012; Bruno et al. 2012; Bruno et al. 2017). These reports suggest that EVs could serve as a novel paracrine therapeutic agent by initiating endogenous repair mechanism(s) in the recipient injured cells and ameliorating the organ damage caused by Cd. Despite the well‐established observation indicating the importance of EVs’ roles in organ repair through paracrine mechanisms, the isolation and evaluation of EVs for in vivo and ex vivo studies remain challenging (van Niel et al. 2018). A classical ultracentrifugation method for EV preparation is time‐consuming, requires a large sample volume, and results in an EV preparation containing carried‐over proteins (such as albumin and immunoglobulin), which mask its efficacy (Greening et al. 2015; Taylor and Shah 2015; Lane et al. 2017).

In the present study, we established an age‐ and sex‐dependent medaka model and showed that it is highly clinically relevant for the study of Cd pathotoxicity, based on the guidelines proposed by the Organization for Economic Co‐operation and Development (OECD) https://www.oecd-ilibrary.org/environment/test-no-203-fish-acute-toxicity-test_9789264069961-en. Cd accumulated in various tissues when medakas were exposed to 4 ppm Cd solution for 4 days. Sex‐dependent accumulation was observed in the liver, kidney and blood, but not in the brain, genitals and intestine. After 4 days of Cd exposure on whole medaka, we evaluated the kidney proximal tubule defects by apical and basolateral membrane markers or dextran‐tetramethylrhodamine (fluoro‐ruby) uptake, and glomerulus defects by histology and ultrastructure. Seven days of Cd exposure triggered bone deformation specifically in aged females. To repair damage caused by Cd poisoning, we developed a protocol for intravenous injection of highly purified hBM‐MSC EVs in our medaka model. The EV protocol that we describe here is effective at repairing tissue damage caused by Cd, and may prove beneficial for organ repair in other diseases.

## Materials and Methods

### Fish maintenance

Medakas (Cab strain) were supplied by NBRP Medaka and raised under standard laboratory conditions at 28°C with a 14 h light/10 h dark cycle (https://shigen.nig.ac.jp/medaka/). All experiments were conducted on 3‐month‐old and 12‐month‐old Cab adult fish. All procedures in this study were approved by the Institutional Animal Care and Use Committee of the University of Oklahoma Health Sciences Center (IACUC protocol No. 18‐044‐CHIB to T.O.) and performed in accordance with the Guide for the Care and Use of Laboratory Animals of the National Institutes of Health.

### Cd exposure medaka model

Seven female or male young (3‐month‐old) or old (12‐month‐old) medaka adults were exposed to Cd for 1, 2, 3 or 4 days in 500 mL system water using 1 L disposable flasks to determine the survival of the fish, following the OECD guidelines (https://www.oecd-ilibrary.org/environment/test-no-203-fish-acute-toxicity-test_9789264069961-en). System water with Cd in the each flask was changed every day.

### Blood and organ Cd concentration measurements

Ten medakas were anesthetized with 0.02% Tricaine®‐S (TRS1, Pentair, Apopka, FL) and caudal fins were cut to collect 1–5 μL blood using 5 μl Drummond micropipettes (1‐000‐0100, Drummond Scientific Co, Broomall, PA). Blood, kidney, liver, heart, genitals, brain, and intestine were harvested and freeze‐dried using the Savant SpeedVac™ Plus Model SC110A (Thermo Fisher). We used the ICP‐MS NexION350 (PerkinElmer, Waltham, MA) mass spectrometer to measure the Cd concentration in blood and organs, according to the manufacturer’s instructions.

### Adult medaka kidney fluoro‐ruby, LTL, a5 and podocalyxin immunohistochemical analyses

Adult medakas were anesthetized by placing fish into a dish containing 0.02% Tricaine‐S for 2 min. The fish were placed ventral side up under the Leica M80 microscope (Leica, Buffalo Grove, IL). A 31G 1.0 cc insulin syringe (BD328438, Thermo Fisher) was used to inject 2 μl of Tetramethylrhodamine 10,000 MW (fluoro‐ruby) (D1817, Thermo Fisher) into the intraperitoneal space, as described previously (McCampbell et al. 2014). The fish were gently returned to the tank system. Fish were euthanized in a dish containing 0.2% Tricaine‐S for 5 min. The fish were immediately dissected to remove kidneys. The kidneys were fixed in 4% paraformaldehyde/phosphate‐buffered saline solution (1xPBS/4% PFA; 15710, Electron Microscopy Sciences, Hatfield, PA) at 4°C. Samples were used for either whole mount or cryo‐embedded in Tissue‐Plus O.C.T. (23‐730‐571, Thermo Fisher) and cut into 10‐micron sections. Fluorescein‐labeled Lotus Tetragonolobus Lectin (LTL) (FL‐1321, Vector Laboratories, Burlingame, CA) was used to label the PT apical membrane, a5 (a5, Developmental Studies Hybridoma Banks, Iowa, IA) was used to label Na^+^/K^+^‐ATPase α‐subunit in the PT basolateral membrane, and DAPI (D1306, Thermo Fisher) was used to label the nuclei (McCampbell et al. 2014). Whole‐mount samples were mounted flat on glass slides for analysis. Podocalyxin immunostaining was performed on 10‐micron cryosections (Ichimura et al. 2012). Whole mount and section samples were imaged with an FV‐1000 confocal laser‐scanning microscope (Olympus, Center Valley, PA).

### Whole‐mount in situ hybridization, histology and TEM analyses

Whole‐mount in situ hybridization was performed as previously described (Ichimura et al. 2012). Medaka *wt1a* was obtained using RT‐PCR from total RNA prepared from 3‐month‐old medaka kidney using the RNAqueous‐4^®^ PCR Kit (AM1914, Thermo Fisher). RT‐PCR was performed using GoScript Reverse Transcription (RT) (A5003, Promega) with p(dN)_6_, followed by a second.

PCR using Green Go Taq PCR (M3001, Promega, Madison, WI). Primers used were: 5’‐GACAGCCTCGAGTGCACTTCTCGGGACAGTTCACAGG‐3’ and 5’‐GCTAGTTCTAGAGAGACAGCTTGAAGTAGCGCTTGTTGC‐3’ (Integrated DNA Technologies, Skokie, IL). The PCR product was digested with *XbaI* and *XhoI* cloned into pBluescript KS^+^ and sequenced with M13 reverse primer. For the *wt1a* antisense RNA probe, pBluescript KS^+^ was linearized *XhoI* and T7 RNA polymerase (R0884, Sigma‐Aldrich, St. Louis, MO) was used to generate the DIG RNA probe. Probe was synthesized using the DIG‐RNA (11277073910, Sigma‐Aldrich) labeling mix according to the manufacturer’s instructions. Alkaline phosphatase‐conjugated anti‐digoxigenin (11093274910, Sigma‐Aldrich) was used to localize the probes. NBT/BCIP (11681451001, Sigma‐Aldrich) was used to produce a blue chromogenic deposit. Whole‐mount samples were imaged with a Leica M165MC microscope (Leica) using the LAS V4.12 program. Four‐micron JB4 sections (00226‐1. Polysciences, Inc., Warrington, PA) were cut with a Leica RN2255 microtome (Leica, Buffalo Grove, IL) and stained with hematoxylin and eosin (HE; 3490, BBC Biomedical, Dallas, TX) to evaluate general structure or with Periodic Acid‐Schiff (PAS; 24200‐1, Polysciences, Inc.). Samples for transmission electron microscopy (TEM) were fixed as previously described (Ichimura et al. 2013). Samples were submitted to Hanaichi UltraStructure Research Institute (Okazaki, Aichi, Japan) for further processing. Ultrathin (80–90 nm) sections were then cut and counterstained with uranyl acetate and lead citrate, and observed using a HITACHI‐H7600 transmission electron microscope at 100 KV (Hitachi, Tokyo, Japan).

### EV purification and specific labeling and IV injection into medaka

Approximately 2 × 10^6^ hBM‐MSCs were seeded and cultured in 150‐mm tissue culture plates with MSC basal medium (ATCC^®^ PCS‐500‐041™, ATCC, Manassas, VA) supplemented with 10% Exo‐FBS (EXO‐FBS‐250A‐1, System Biosciences [SBI], Palo Alto, CA), 2 mmol/L Glutamax (35050061, Thermo Fisher), and 100 units/mL penicillin and 100 units/mL streptomycin (15140122, Thermo Fisher). Cells were incubated in a 37°C incubator with 5% CO_2_ for 72 h until EVs were harvested from 20 mL of media using the methods described below. EVs were isolated using ultracentrifugation (UC) and ExoQuick‐TC ULTRA (EQULTRA‐20TC‐1, SBI), described in detail below. For UC, cells and cell debris were removed. The sample was centrifuged using an Optima XP‐MAX ultracentrifuge (Beckman‐Coulter, Brea, CA) at 10,000*g* for 30 min at 4°C, followed by a second spin at 100,000*g* for 60 min (4°C) to pellet the EV fraction. The resulting pellet was washed once with 1X PBS at 100,000*g* for 60 min (4°C). The pellet was used for a downstream labeling assay using ExoGlow‐Protein labeling reagent (EXOGP100A‐1, SBI). For ExoQuick‐ULTRA, isolation of EVs was performed according to the manufacturer’s instructions. Briefly, 10 mL of the culture medium was mixed with 2 mL of ExoQuick‐TC and incubated 16 h at 4°C. The next day, the admixture was centrifuged at 3000*g* for 15 min at 4°C to pellet the EVs. The pellet was resuspended in 200 µL of Buffer B and placed into a column containing resin to purify residual protein and protein aggregates. EVs were eluted by spinning at 1000*g* for 30 sec in a table‐top centrifuge. EVs were labeled using an ExoGlow‐Protein EV labeling kit (EXOGP100A‐1, SBI) according to the manufacturer’s instructions. Pellets were resuspended in 100 μL of 1X PBS. Total protein concentration was measured using the Qubit Protein Assay Kit (Q33211, Thermo Fisher). For IV injection, we used 4 × 10^7^ EV per medaka using a 2 μl injection volume. Please see Supplementary Information for a description of how we conformed to the 2018 new ISEV guidelines for characterization of extracellular vesicles (Théry et al. 2018).

### Fluorescence nanoparticle tracking analysis (fNTA) for EVs

Two microliters of EVs purified using the ExoQuick‐TC ULTRA or UC method were labeled using the ExoGlow‐NTA Fluorescent Labeling Kit (EXONTA200A‐1, SBI), according to the manufacturer’s recommended protocol. Labeled EVs were diluted by a factor of 10–1000 (depending on the isolation method) in a final volume of 300 μL for NTA using the NanoSight LM10 fitted with a 488‐nm wavelength laser and 500‐nm long‐pass filter (Malvern Instruments, Malvern, UK). Both light scattering and fluorescence modes were employed to detect particle counts/mL and size distribution of particles in solution. Particle counts/mL and size distribution of both modes were overlaid to generate light scattering and fluorescent counts to determine the overall fraction of EVs in the solution that were labeled to generate EV‐specific particle concentration and sizing data.

### Whole‐mount bone staining

Whole‐mount bone staining of medaka was performed as previously described, with the modifications described below (Sakata‐Haga et al. 2018). Tricaine‐S‐anesthetized medaka were immersed in freshly prepared fixative (5% Formalin/5% Triton X‐100/1% KOH) (HT501128, T8787, 221473, Sigma‐Aldrich) for 24 h at 42°C with rotation. Internal organs were removed without damaging ribs and skin using a cotton swab, washed in tap water, and immersed in alizarin red S staining solution (0.05% alizarin red S, 20% ethylene glycol, 1% KOH; A5533, 324558, 221473, Sigma‐Aldrich) for 30 min at room temperature; washed with alizarin red S washing solution (1% KOH, 20% Tween‐20 [221473, P9416, Sigma‐Aldrich]); and gently rotated at 42°C for 16 h. Scales were removed from skin using a cotton swab, washed in tap water and immersed in alizarin red S staining solution for 30 min at room temperature, then gently washed with alizarin red S washing solution and rotated at 42°C for 16 h. Specimens were transferred to 70% glycerol 35 mL (G33‐1, Thermo Fisher) +21% EtOH (111000200, PHARMCO‐AAPER) for storage. Samples were imaged with a Leica M165MC microscope (Leica) using the LAS V4.12 program.

### Statistical analysis

All statistical analyses were performed using GraphPad Prism 8.00 for Windows (GraphPad Software, La Jolla, CA). Variance is displayed as SEM, as noted in the figure legends. Differences were considered to be statistically significant at *P *<* *0.05. Ordinary two‐way ANOVAs were used to test for interaction between Cd treatment and sex in Cd accumulation in tissue, and for the interaction between Cd concentration and sex in PT fluoro‐ruby uptake. Survival curve comparisons were performed using GraphPad Prism 8.00 for Windows, followed by Mantel–Cox log‐rank and Gehan–Breslow–Wilcoxon tests.

## Results

### Cd exposure conditions to study the xenobiotic pathotoxicity in adult medaka

**Figure 1:**
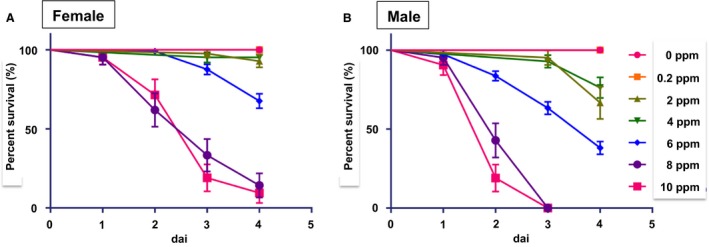
Survival curves of Cd‐exposed adult female and male medaka. Medaka were exposed to 0–10 ppm Cd for 4 days (dai) at 28°C. (A) Female. (B) Male.

Recently all‐male mouse and rat models have confirmed that exposure to Cd alone can mimic some aspects of pathotoxic phenotype of Cd poisoning reported in humans (Prozialeck et al. 2007; Edwards and Prozialeck 2009; Prozialeck et al. 2009a; Prozialeck et al. 2009b; Yamanobe et al. 2015; Prozialeck et al. 2016). However, since Cd was applied at high concentrations in these studies, the results lacked clinical relevance by missing the effect of lower concentrations and chronic Cd exposure, as well as age‐ and sex‐dependent toxicity (Prozialeck et al. 2007; Edwards and Prozialeck 2009; Prozialeck et al. 2009a; Prozialeck et al. 2009b; Yamanobe et al. 2015; Prozialeck et al. 2016). Since adult medakas have historically been used for environmental toxicology, they provided an outstanding experimental paradigm (Wittbrodt et al. 2002; Wessely and Obara 2008; Walter and Obara 2015). In the medaka model, a wide range of genetic and pharmacological tools are available that can be used to delineate the cellular and molecular mechanisms that regulate renal degeneration and regeneration. We set up a Cd exposure protocol based on the guidelines proposed by OECD and created 3‐month‐old female and male medaka for testing 0, 0.2, 2, 4, 6, 8 and 10 ppm Cd exposure for 1–4 days (dai) (Fig. 1).

Females exposed to 0, 0.2, 2, and 4 ppm Cd had a 100% survival rate at 4 days. Females receiving the 6 ppm dose declined after 3 days, while females receiving the 8 and 10 ppm doses declined after day 2, and only 15% survived at 4 days of exposure (Fig. 1A). Males exposed to 0 and 0.2 ppm Cd displayed 100% survival at 4 days. Survival in males declined to 70–80% at 4 days of exposure with doses of 2 and 4 ppm, and decreased to 84%, 63%, and 38%, respectively, after 2, 3, and 4 days of exposure to 6 ppm Cd. In addition, 19–43% of males survived after 2 days of exposure to 8 and 10 ppm Cd, and all males died after 4 days of exposure to 8 and 10 ppm Cd (Fig. 1B). Based on these data, concentrations that kill 50% of the fish (LC_50_, lethal concentration, 50%) were determined to be 7.5 ppm for females and 5.5 ppm for males at 4 days of exposure. Therefore, we decided to use Cd doses of 0.2, 1, and 2 ppm for 4 days to evaluate kidney pathology and function.

**Figure 2:**
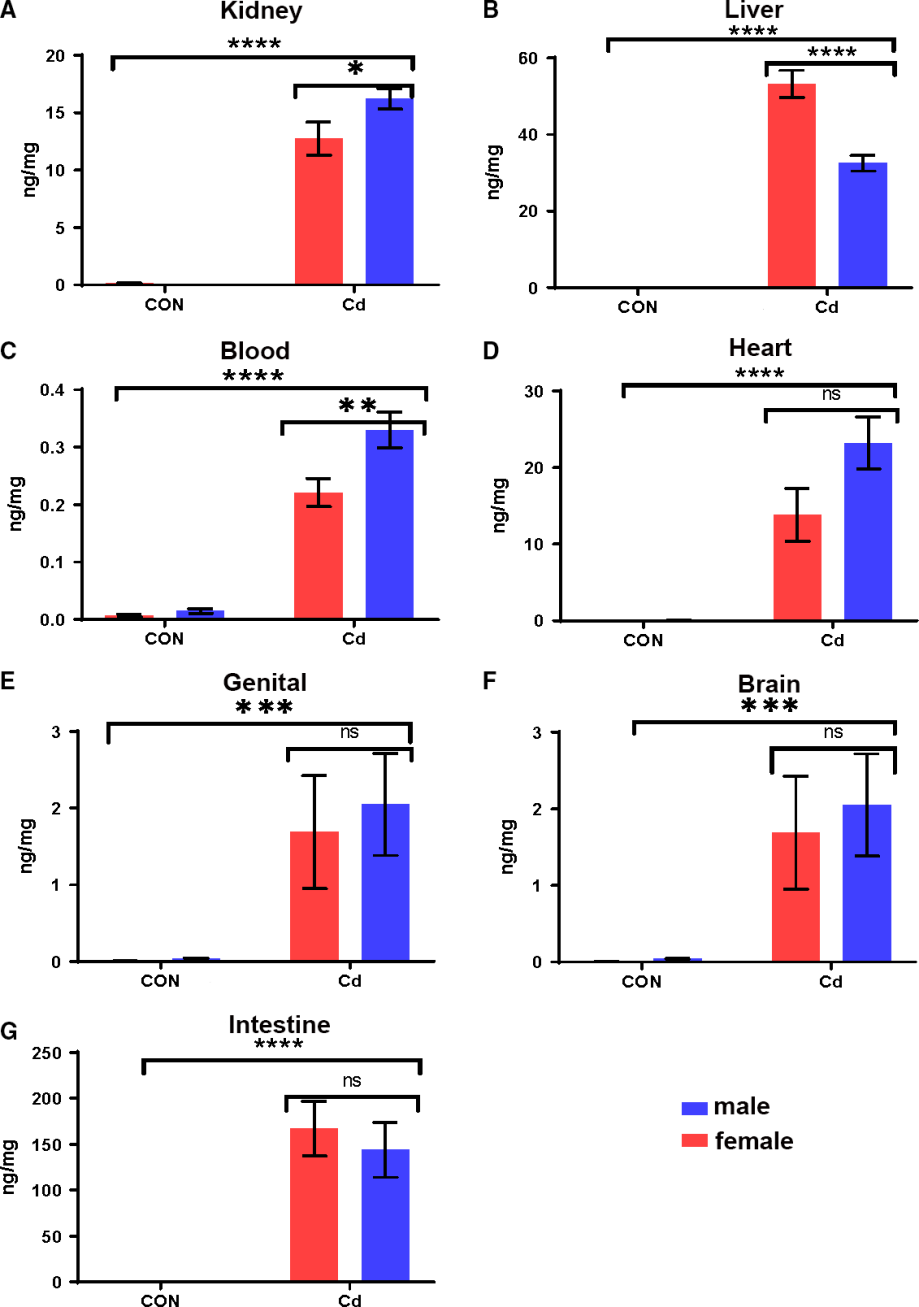
Cd accumulation in the blood and organs when medaka were exposed to 4 ppm Cd for 4 days. Two‐way ANOVA analysis showed interaction between sex and Cd treatment in kidney (A), liver (B), and blood (C), but not in heart (D), genitals (E), brain (F), and intestine (G). Analyzed using two‐way factorial ANOVA, followed by Sidak post hoc test with GraphPad Prism software 8.0. *P <* *0.5; **P <* *0.01; ***P <* *0.001; ****P <* *0.0001. 0 ppm Cd exposure (CON), and 4 ppm Cd exposure (Cd).

Figure 2 summarizes an analysis of inductively coupled plasma mass spectrometry (ICP‐MS) to evaluate Cd accumulation; these include the Cd accumulation normalized to tissue weights. Blood (0.221 ng/mg in females and 0.330 ng/mg in males), kidney (12.731 ng/mg in females and 16.212 ng/mg in males), liver (53.205 ng/mg in females and 32.500 ng/mg in males), intestine (166.989 ng/mg in females and 143.895 ng/mg in males), heart (13.824 ng/mg in females and 23.210 ng/mg in males), brain (0.130 ng/mg in females and 0.241 ng/mg in males), and genitals (1.689 ng/mg in females and 2.052 ng/mg in males).

Two‐way ANOVA (Cd treatment, sex) showed an interaction between treatment and sex for Cd accumulation in the liver, kidney, and blood, but not in the brain, genitals, or intestine. In the liver, there was a significant main effect for Cd treatment (*F*_1,36_ = 433.1, *P *<* *0.0001), sex (*F*_1,36_ = 25.25, *P *<* *0.001), and interaction between factors (*F*_1,36_ = 25.25, *P *<* *0.0001). In the kidney, there was a significant main effect for treatment (*F*_1,36_ = 282.4, *P *<* *0.0001), a nonsignificant effect for sex (*F*_1,36_ = 3.957, *P *=* *0.0538), and a significant interaction between factors (*F*_1,36_ = 4.348. *P* = 0.042). In the blood, Cd treatment was the main effect (*F*_1,36_ = 175.3, *P *<* *0.0001), sex was significant (*F*_1,36_ = 8.575, *P *=* *0.0538), and the interactions between the factors were significant (*F*_1,36_ = 6.390, *P *=* *0.442). The heart, genitals, brain, and intestine showed a significant effect for treatment (heart *F*_1,36_ = 58.2, *P *<* *0.0001; genital and brain *F* = 13.74, *P *=* *0.0007; intestine *F* = 54.17, *P *<* *0.0001), but not for sex (heart *F* = 3.775, *P *=* *0.0599; genital *F* = 0.01589, *P *=* *0.6925; brain *F* = 0.01589, *P *=* *0.6925; intestine *F* = 0.2992, *P *=* *0.5878) or interaction between factors (heart *F* = 3.742, *P *=* *0.0610; genital *F* = 0.1091, *P *=* *0.7430; brain *F* = 0.1091, *P *=* *0.743; intestine *F* = 0.3002, *P *=* *0.5871) (Fig. 2 and Table 1). These findings confirmed (1) that Cd accumulated in various tissues when adult medakas were exposed to 4 ppm Cd solution for 4 days, and (2) that there was sex‐dependent accumulation in the liver, kidney and blood, but not in the brain, genitals and intestine.

**Table 1:** Two‐way ANOVA analysis between Cd treatment and sex.

Liver				
Source of variation	% of total variation	*P* value	*P* value summary	Significant
Interaction	4.908	*P *<* *0.0001	****	Yes
Treatment	83.31	*P *<* *0.0001	****	Yes
Sex	4.858	*P *<* *0.0001	****	Yes

ANOVA table	SS	df	MS	*F* (DFn, DFd)	*P* value
Interaction	1,077	1	1,077	*F* (1, 36) = 25.51	*P *<* *0.0001
Treatment	18,286	1	18,286	*F* (1, 36) = 433.1	*P *<* *0.0001
Sex	1,066	1	1,066	*F* (1, 36) = 25.25	*P *<* *0.0001
Residual	1,520	36	42.22		

Kidney				
Source of variation	% of total variation	*P* value	*P* value summary	Significant
Interaction	1.331	*P *=* *0.0442	*	Yes
Treatment	86.43	*P *<* *0.0001	****	Yes
Sex	1.217	*P *=* *0.0538	ns	No

ANOVA table	SS	df	MS	*F* (DFn, DFd)	*P* value
Interaction	31.67	1	31.67	*F* (1, 36) = 4.348	*P *=* *0.0442
Treatment	2,057	1	2,057	*F* (1, 36) = 282.4	*P *<* *0.0001
Sex	28.95	1	28.95	*F* (1, 36) = 3.975	*P *=* *0.0538
Residual	262.2	36	7.284		

Blood				
Source of variation	% of total variation	*P* value	*P* value summary	Significant
Interaction	2.824	*P *=* *0.016	*	Yes
Treatment	77.48	*P *<* *0.0001	****	Yes
Sex	3.79	*P *=* *0.0059	**	Yes

ANOVA table	SS	df	MS	*F* (DFn, DFd)	*P* value
Interaction	0.0255	1	0.0255	*F* (1, 36) = 6.390	*P *=* *0.016
Treatment	0.6996	1	0.6996	*F* (1, 36) = 175.3	*P *<* *0.0001
Sex	0.03422	1	0.03422	*F* (1, 36) = 8.575	*P *=* *0.0059
Residual	0.1437	36	0.003991		

Heart				
Source of variation	% of total variation	*P* value	*P* value summary	Significant
Interaction	3.679	*P *=* *0.016	ns	No
Treatment	57.22	*P *<* *0.0001	****	Yes
Sex	3.712	*P *=* *0.0599	ns	No

ANOVA table	SS	df	MS	*F* (DFn, DFd)	*P* value
Interaction	219.3	1	219.3	*F* (1, 36) = 3.742	*P *=* *0.0610
Treatment	3,410	1	3,410	*F* (1, 36) = 58.20	*P *<* *0.0001
Sex	221.2	1	221.2	*F* (1, 36) = 3.775	*P *=* *0.0599
Residual	2,109	36	58.6		

Genitals				
Source of variation	% of Total variation	*P* value	*P* value summary	Significant
Interaction	0.2183	*P *=* *0.743	ns	No
Treatment	27.47	*P *=* *0.0007	***	Yes
Sex	0.3178	*P *=* *0.6925	ns	No

ANOVA table	SS	df	MS	*F* (DFn, DFd)	*P* value
Interaction	0.2706	1	0.2706	*F* (1, 36) = 0.1091	*P *=* *0.7430
Treatment	34.06	1	34.06	*F* (1, 36) = 13.74	*P *<* *0.0007
Sex	0.394	1	0.394	*F* (1, 36) = 0.1589	*P *=* *0.06925
Residual	89.26	36	2.479		

Brain				
Source of variation	% of total variation	*P* value	*P* value summary	Significant
Interaction	0.2183	*P *=* *0.743	ns	No
Treatment	27.47	*P *=* *0.0007	***	Yes
Sex	0.3178	*P *=* *0.6925	ns	No

ANOVA table	SS	df	MS	*F* (DFn, DFd)	*P* value
Interaction	0.2706	1	0.2706	*F* (1, 36) = 0.1091	*P *=* *0.7430
Treatment	34.06	1	34.06	*F* (1, 36) = 13.74	*P *=* *0.0007
Sex	0.394	1	0.394	*F* (1, 36) = 0.1589	*P *=* *0.6925
Residual	89.26	36	2.479		

Intestine				
Source of variation	% of total variation	*P* value	*P* value summary	Significant
Interaction	0.3307	*P *=* *0.5871	ns	No
Treatment	59.68	*P *<* *0.0001	***	Yes
Sex	0.3296	*P *=* *0.5878	ns	No

ANOVA table	SS	df	MS	*F* (DFn, DFd)	*P* value
Interaction	1,336	1	1,336	*F* (1, 36) = 0.3002	*P *=* *0.5871
Treatment	241,029	1	241,029	*F* (1, 36) = 54.17	*P *<* *0.0001
Sex	1,331	1	1,331	*F* (1, 36) = 0.2992	*P *=* *0.5878
Residual	160,175	36	4,449		

Data tables from two‐way ANOVA analysis to test the interaction between sex and Cd treatment in tissue‐specific Cd accumulation. alpha = 0.05.

### Pathotoxic phenotypes in Cd‐exposed adult medaka kidneys

The kidney is the primary target of Cd toxicity upon chronic low‐level exposure to humans. Cd causes a generalized dysfunction of the proximal tubule (PT) that is characterized by polyuria and proteinuria (Lane et al. 2015; Aoshima 2016). Much attention has focused on the identification of urinary biomarkers of the early stages of Cd nephrotoxicity, such as metallothionein (Dorian et al. 1992; Nordberg et al. 2007; Onodera et al. 2012). Metallothionein is a specific metal‐binding protein that plays a role in concentrating Cd in the PT epithelium (Dorian et al. 1992; Nordberg et al. 2007; Onodera et al. 2012). Although the adult medaka kidney has fewer nephrons, the structural units of the kidney, and a less complex overall structure, the basic renal structure and function are largely conserved between medaka and mammalian kidney (20022; Wessely and Obara 2008; Walter and Obara 2015). These similarities have enabled medaka to be an effective research tool to model numerous kidney diseases, in addition to the substantial list of human conditions that can be investigated using medaka (Wittbrodt et al. 2002; Wessely and Obara 2008; Walter and Obara 2015).

**Figure 3:**
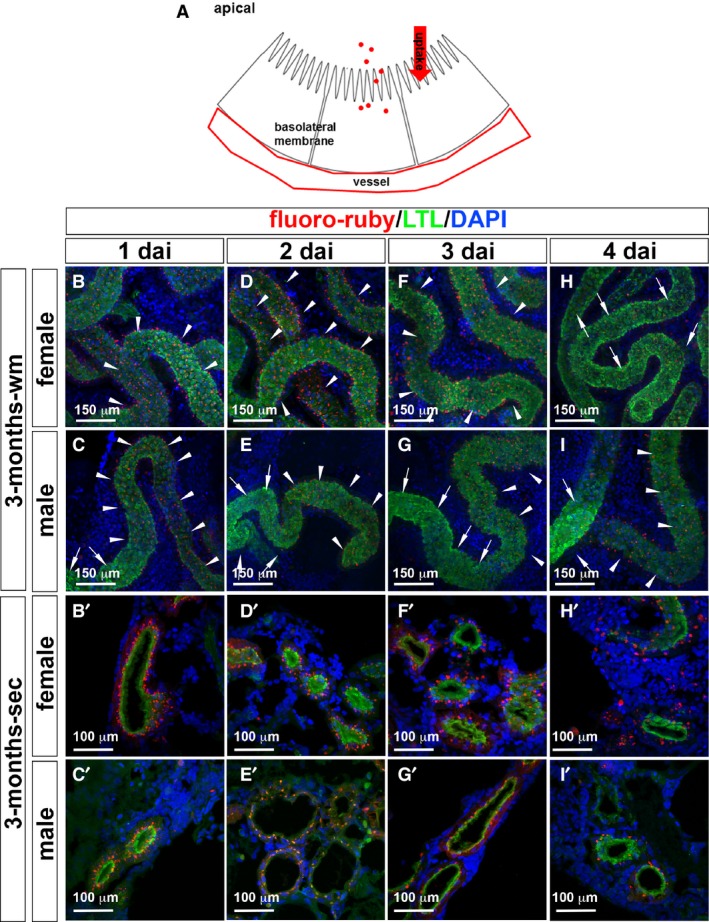
Dynamics of fluoro‐ruby uptake in the proximal tubules of medaka by flat‐mount and cryosection immunohistochemical analyses. (A) Diagram of the fluoro‐ruby uptake from the proximal tubule apical membrane. Flat whole‐mount (wm) adult medaka kidney (B–I) and cryosection (sec) (B’–I’). Fluoro‐ruby‐uptake (red), LTL (green), and DAPI (blue).

Since the effects of Cd on PT were observed (Aoshima 2016), we examined the effects of Cd in the medaka PT. To assess PT function, we performed fluoro‐ruby (dextran‐tetramethylrhodamine, MW = 10,000) uptake assays (Fig. 3). PT apical membrane epithelium will incorporate fluoro‐ruby present in the circulation, which can be used to assess endocytosis uptake by the PT apical membrane (Fig. 3A) (McCampbell et al. 2014). To optimize the conditions for 3‐month‐old female and male medaka, we dissected adult kidneys after 1, 2, 3, or 4 days of intraperitoneal (IP) injection with fluoro‐ruby. To further confirm that the PT took up fluoro‐ruby, the whole mount and single nephrons were colabeled with fluorescence‐tagged lectin Lotus Tetragonolobus (LTL), a vertebrate PT apical membrane marker. Fluoro‐ruby showed overlap with LTL localized to the apical region of PT (McCampbell et al. 2014) (Fig. 3B–I and B’–I’), confirming that EVs were taken up into PT. Next, the colabeling of nephron tubules with LTL and fluoro‐ruby was observed in immunohistochemical cryosections, and LTL was noted in a thick band at the apical surface of the tubular epithelium (Fig. 3B’–I’). Notably, stained tubules were double‐positive for LTL and fluoro‐ruby in both females and males (Fig. 3B, C, B’ and C’). The uptake was well observed from 1 dai onwards gradually decreased in a time‐dependent manner (Fig. 3D–I and D’–I’). We decided to observe fluoro‐ruby uptake into PT at 1 dai. We note that the above protocol to evaluate fluoro‐ruby uptake can be utilized for the characterization of other medaka renal disease models.

**Figure 4:**
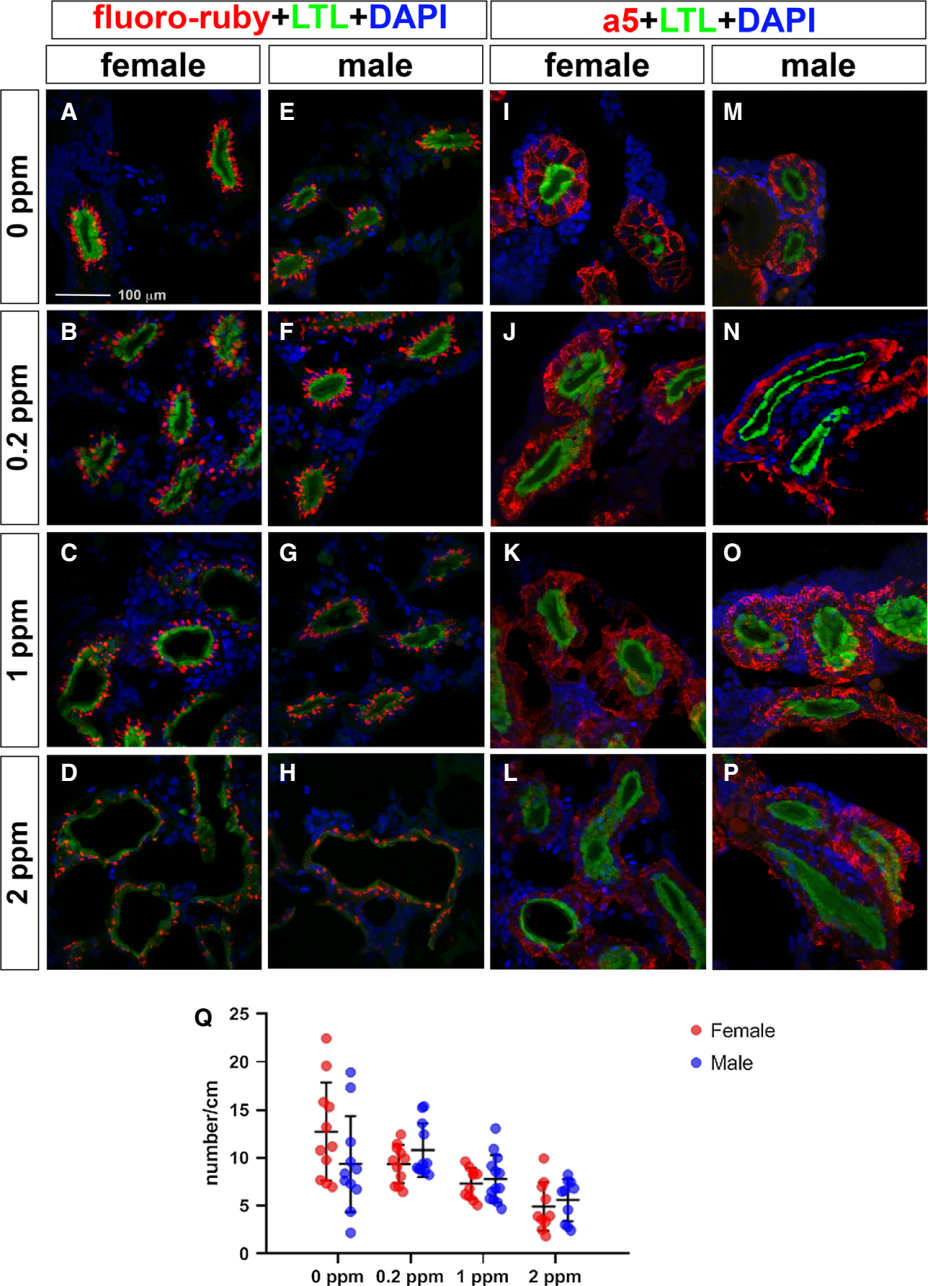
LTL, a5 staining, and fluoro‐ruby uptake in the proximal tubules in Cd‐exposed medaka. (A–P) Immunofluorescence images using kidney cryosections. (A–H) LTL labeled the proximal tubule apical membrane (green), fluoro‐ruby (red), and DAPI labeled nuclei (blue). (I–P) LTL labeled the proximal tubule apical membrane (green); Na^+^, K^+^‐ATPase alpha subunit (a5) labeled the proximal tubule basolateral membrane (red); and DAPI labeled the nuclei (blue). The scale bar indicates 100 mm. (Q) Distribution of fluoro‐ruby uptake per cm proximal tubule in female (red) and male (blue) medaka treated with Cd. Mean and SD indicated by bars.

We used fluoro‐ruby, LTL, and Na^+^/K^+^‐ATPase α‐subunit (a5) basolateral membrane markers to evaluate the pathotoxic damage caused by Cd at lower concentrations (0.2–2.0 ppm) at a short exposure time of 4 days. The uptake of fluoro‐ruby, LTL and a5 was affected at 0.2 ppm in females and at 1.0 ppm in males (Fig. 4A–Q). Two‐way ANOVA analysis showed that Cd concentration had a significant main effect on fluoro‐ruby uptake (*F*_3,78_ = 13.29, *P *<* *0.0001), but that sex and the interaction between sex and Cd concentration were not significant (sex, *F* = 0.06037; interaction, *F* = 2.307, *P *=* *0.0830). We did a multiple comparison test to compare changes in uptake at different levels of Cd. In females, there was a slight, nonsignificant increase in uptake at 0.2 ppm (mean of 9.346 for control, 12.74 for 0.2 ppm) compared with control. At higher levels of Cd, the uptake decreased. Only the 2 ppm dose showed a significant decrease in uptake (mean of 4.928 compared with 9.346 for control; Fig. 4Q and Table 2).

**Table 2:** Brown–Forsythe and Welch ANOVA test (No assumption of equal SDs between columns)

Female
Number of families: 1
Number of comparisons per family: 6
Alpha: 0.05

Tamhane's T2 multiple comparisons test	Mean Diff.	95% CI of diff.	Significance	Summary	Adjusted
0 ppm versus 0.2 ppm	−3.397	−8.513 to 1.719	No	ns	0.3121
0 ppm versus 1 ppm	2.014	−0.2925 to 4.321	No	ns	0.1089
0 ppm versus 2 ppm	4.418	1.439 to 7.398	Yes	**	0.0023
0.2 ppm versus 1 ppm	5.411	0.3319 to 10.49	Yes	*	0.0344
0.2 ppm versus 2 ppm	7.815	2.560 to 13.07	Yes	**	0.0025
1 ppm versus 2 ppm	2.404	−0.4631 to 5.271	No	ns	0.1293

Test details	Mean 1	Mean 2	Mean Diff.	SE of Diff.	*n*1	*n*2	T	df
0 ppm versus 0.2 ppm	9.346	12.74	−3.397	1.652	11	11	2.056	12.97
0 ppm versus 1 ppm	9.346	7.332	2.014	0.785	11	10	2.566	18.75
0 ppm versus 2 ppm	9.346	4.928	4.418	1.002	11	10	4.408	17.07
0.2 ppm versus 1 ppm	12.74	7.332	5.411	1.62	11	10	3.34	12.12
0.2 ppm versus 2 ppm	12.74	4.928	7.815	1.736	11	10	4.502	14.95
1 ppm versus 2 ppm	7.332	4.928	2.404	0.9492	10	10	2.533	15.18

Male
Number of families: 1
Number of comparisons per family: 6
Alpha: 0.05

Tamhan's T2 multiple comparisons test	Mean Diff.	95% CI of diff.	Significance	Summary	Adjusted
0 ppm versus 0.2 ppm	1.409	−4.351 to 7.169	No	Ns	0.9763
0 ppm versus 1 ppm	2.995	−0.1580 to 6.149	No	Ns	0.0687
0 ppm versus 2 ppm	5.212	2.015 to 8.409	Yes	***	0.0008
0.2 ppm versus 1 ppm	1.587	−4.082 to 7.256	No	Ns	0.9518
0.2 ppm versus 2 ppm	3.804	−1.876 to 9.484	No	Ns	0.2977
1 ppm versus 2 ppm	2.217	−0.6200 to 5.054	No	Ns	0.1866

Test details	Mean 1	Mean 2	Mean Diff.	SE of Diff.	*n*1	*n*2	T	df
0 ppm versus 0.2 ppm	10.82	9.41	1.409	1.869	11	10	0.7539	13.34
0 ppm versus 1 ppm	10.82	7.823	2.995	1.082	11	13	2.767	20.3
0 ppm versus 2 ppm	10.82	5.606	5.212	1.087	11	10	4.794	18.66
0.2 ppm versus 1 ppm	9.41	7.823	1.587	1.806	11	13	0.8787	12.03
0.2 ppm versus 2 ppm	9.41	5.606	3.804	1.808	11	10	2.103	12.01
1 ppm versus 2 ppm	7.823	5.606	2.217	0.975	13	10	2.274	20.5

One‐way ANOVA analysis to test the difference in fluoro‐ruby uptake between each Cd dose in either females or males. In both sexes, fluoro‐ruby uptake was decreased when the Cd concentration was 2 ppm. The Brown–Forsythe and Welch ANOVA tests were used because the SDs were not the same in each Cd dose. Tamhane’s multiple comparisons test was used to compute individual variances for each comparison. Difference between columns (Mean Diff.), 95% Confidence Interval of difference (95% CI of diff.) Not significant (ns), * for *P *<* *0.05, ** for *P *<* *0.01, *** for *P *<* *0.001, **** for *P *<* *0.0001.

**Figure 5:**
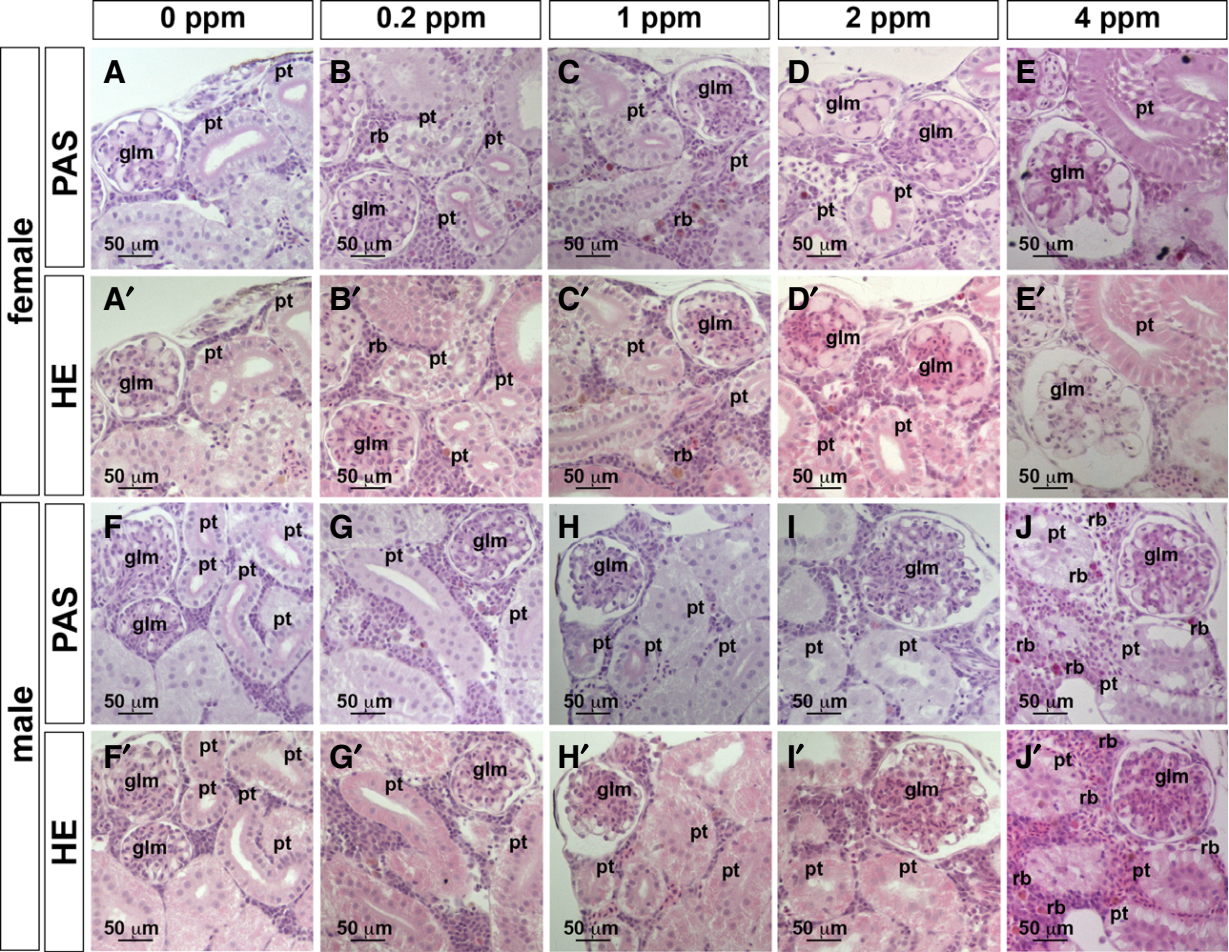
HE and PAS staining of JB4 sections in the adult medaka kidney after 4 days of Cd exposure. (A, A’, F, F’) 0 ppm Cd; (B, B’, G, G’) 0.2 ppm Cd; (C, C’, H, H’) 1 ppm Cd; (D, D’, I, I’), 2 ppm Cd; (E, E’, J, J’), 4 ppm Cd. (A–E, A’–E’) female, (F–J, F’–J’) male. HE‐stained (A–J), PAS‐stained (A’–J’) JB4 sections. Glomeruli (glm) and proximal tubule (pt). The scale bars indicate 50 μm.

Functional disruption of PT has been reported in rat and mouse models of Cd toxicity (Prozialeck and Lamar 1998; Prozialeck et al. 2003). Using this low‐dose, 4‐day exposure of Cd, we examined nephron structure with histological stains of JB4‐embedded kidney sections. As expected, the glomerulus and PT were histologically normal at 0 ppm Cd in both female and male kidneys (Fig. 5A). In the female medaka, the misalignment of apical and basolateral membranes of PT was observed at 0.2–4 ppm (Fig. 5B–E and B’–E’), red blood cell leakage was observed at 2–4 ppm of Cd exposure (Fig. 5D, E, D’ and E’), and enlarged glomeruli were also observed with PAS‐positive mesangial matrix expansion at 2–4 ppm (Fig. 5D, E, D’ and E’), further resulting in thinning of PT apical and basolateral membranes and clearing space in the intracellular region at 4 ppm (Fig. 5E and E’). The red blood cell leakage was observed before the glomerular structure was affected, so the red blood cells in the kidney may reflect loss of vascular integrity and eventual loss of glomerular barrier function.

**Figure 6:**
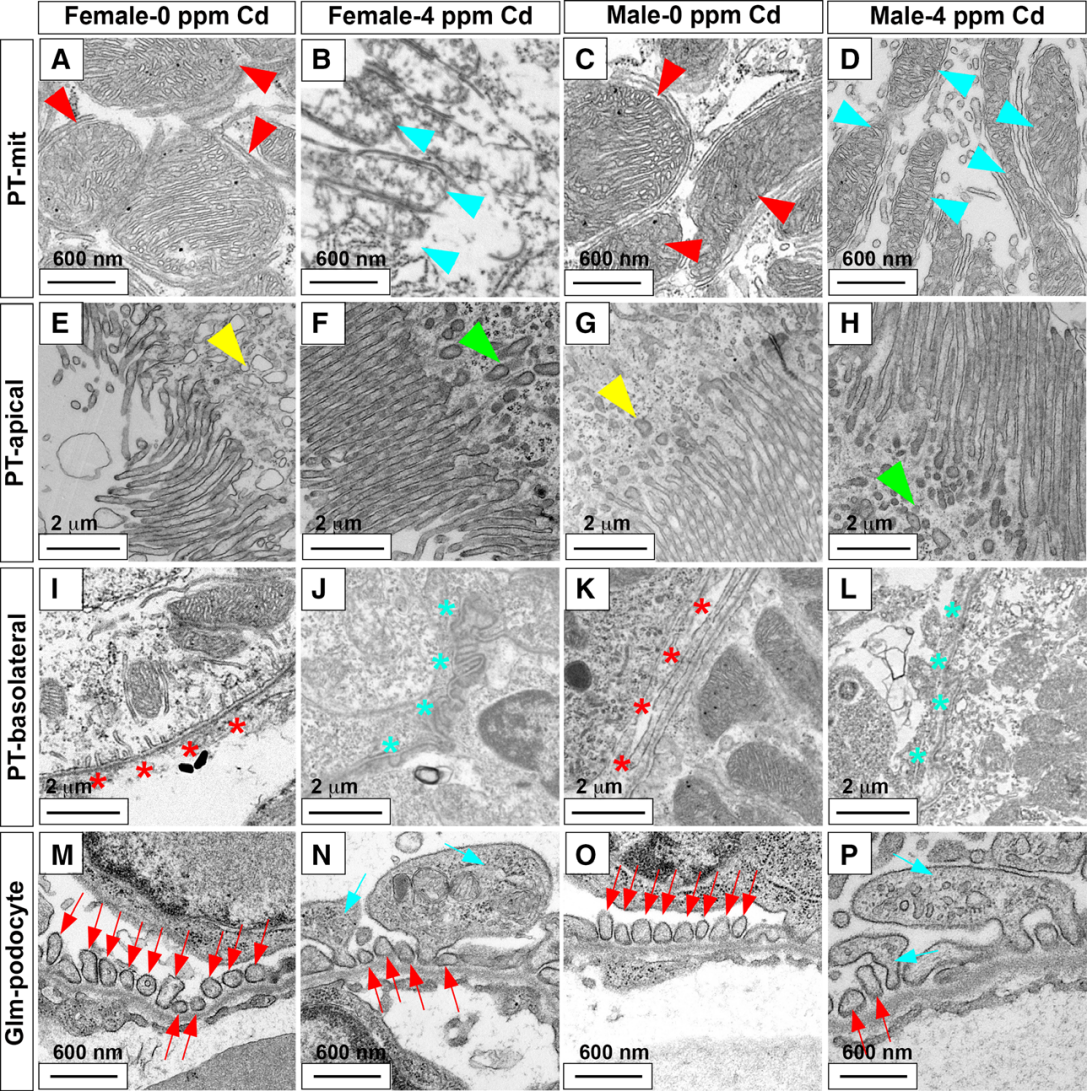
Transmission electron microscope images of proximal tubules and glomeruli in Cd‐exposed medaka. Normal proximal tubule mitochondria (PT‐mit) (red arrowhead; A, C), normal PT apical membrane (PT‐apical) vesicle (yellow arrowhead; E, G), normal PT basolateral membrane (PT‐basolateral; red asterisks; I, K), normal glomeruli podocytes (Glm‐podocyte; red arrow; M, O), damaged PT mitochondria (light blue arrowhead; B, D), abnormal PT apical vesicles (green arrowhead; F, G), abnormal PT basolateral (light blue asterisk; J, L), enlarged podocyte foot process with apoptotic vesicles (light blue arrow; N, P). Female 0 ppm Cd (A, E, I, M), female 4 ppm Cd (B, F, J, N), male 0 ppm Cd (C, G, K, O), male 4 ppm Cd (D, H, L, P). The scale bar indicates 600 nm (A–D, M–P) or 2 μm (E–L).

In the male medaka, in contrast, the misalignment of PT apical and basolateral membranes started at 1–4 ppm (Fig. 5H–J and H’–J’), the leakage of red blood cells at 4 ppm (Fig. 5J and J’), enlargement of glomerular structure at 2–4 ppm (Fig. 5I, J, I’ and J’), and thinning of PT apical and basolateral membranes and intracellular appearance of open space at 4 ppm (Fig. 5J and J’). We examined the cellular ultrastructure using transmission electron microscopy (TEM) to determine whether Cd exposure altered cellular architecture and organelles in the nephron (Ichimura et al. 2012). Surprisingly, significant disassembly of PT mitochondria was observed in females, while only minimal PT mitochondrial changes were seen in males (Fig. 6A–D).

**Figure 7:**
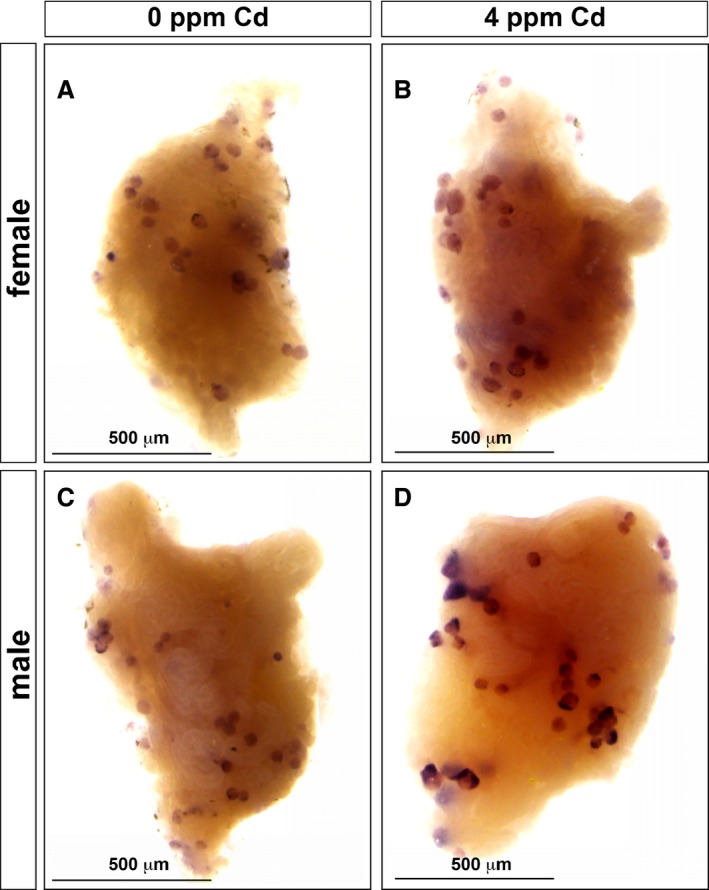
*wt1a* expression in the glomerular podocytes was not affected in Cd‐exposed medaka. (A–D) External views of the whole kidney stained by *wt1a* in situ hybridization. (A, C) Three‐month‐old medaka exposed to 0 ppm Cd. (B, D) 4 ppm Cd‐treated kidney. (A, B) Female. (C, D) Male. The scale bars indicate 500 μm.

**Figure 8:**
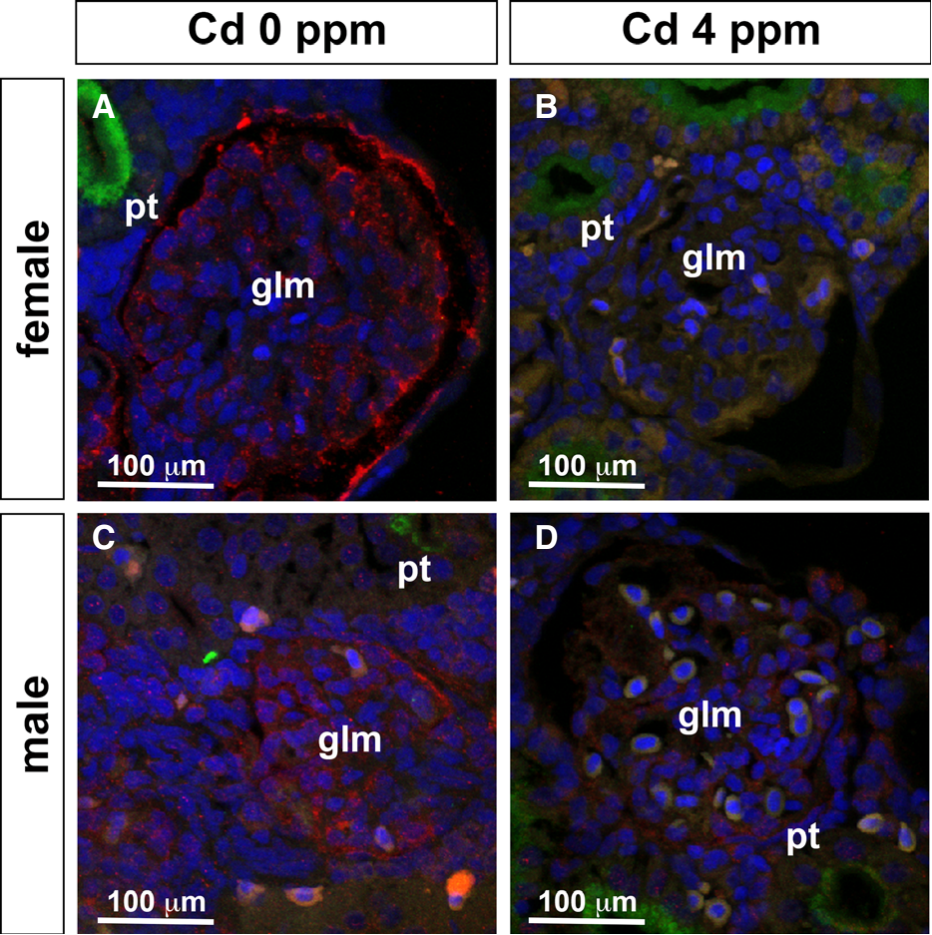
Podocalyxin expression in the glomerular podocytes is reduced in Cd‐exposed female and male medaka. (A–D) Immunofluorescence images using kidney cryosections. (A, C) 0 ppm Cd, (B, D) 4 ppm Cd for 4 days exposure; (A, B) female, (C, D) male. (A–D) Podocalyxin (red), LTL (green), DAPI (blue). Glomerulus (glm) and proximal tubule (pt). The scale bars indicate 100 μm.

In contrast to the sex‐dependent mitochondrial changes, other ultrastructural changes were observed in both sexes. These changes included misshapen apical membranes (Fig. 6E–H), misaligned basolateral membranes (Fig. 6I–L), and enlarged glomerular podocytes (Fig. 6M–P). Abnormal glomerular and vessel rupture resulting in the presence of blood cells in the kidney and enlarged glomerular podocytes was observed only at higher Cd exposure, suggesting that these defects followed the pathotoxic effects on PT epithelium and mitochondria. Although the 4‐day exposure of Cd at 4 ppm resulted in enlarged podocytes in females and males, expression of *wt1a* gene, a podocyte marker, was unaffected (Fig. 7). Expression of Podocalyxin protein, a podocyte surface marker, was absent from the glomerular podocytes of both females and males (Fig. 8).

### Human BM‐MSC EVs repaired kidney and bone defects caused by Cd exposure, and promoted survival

**Figure 9:**
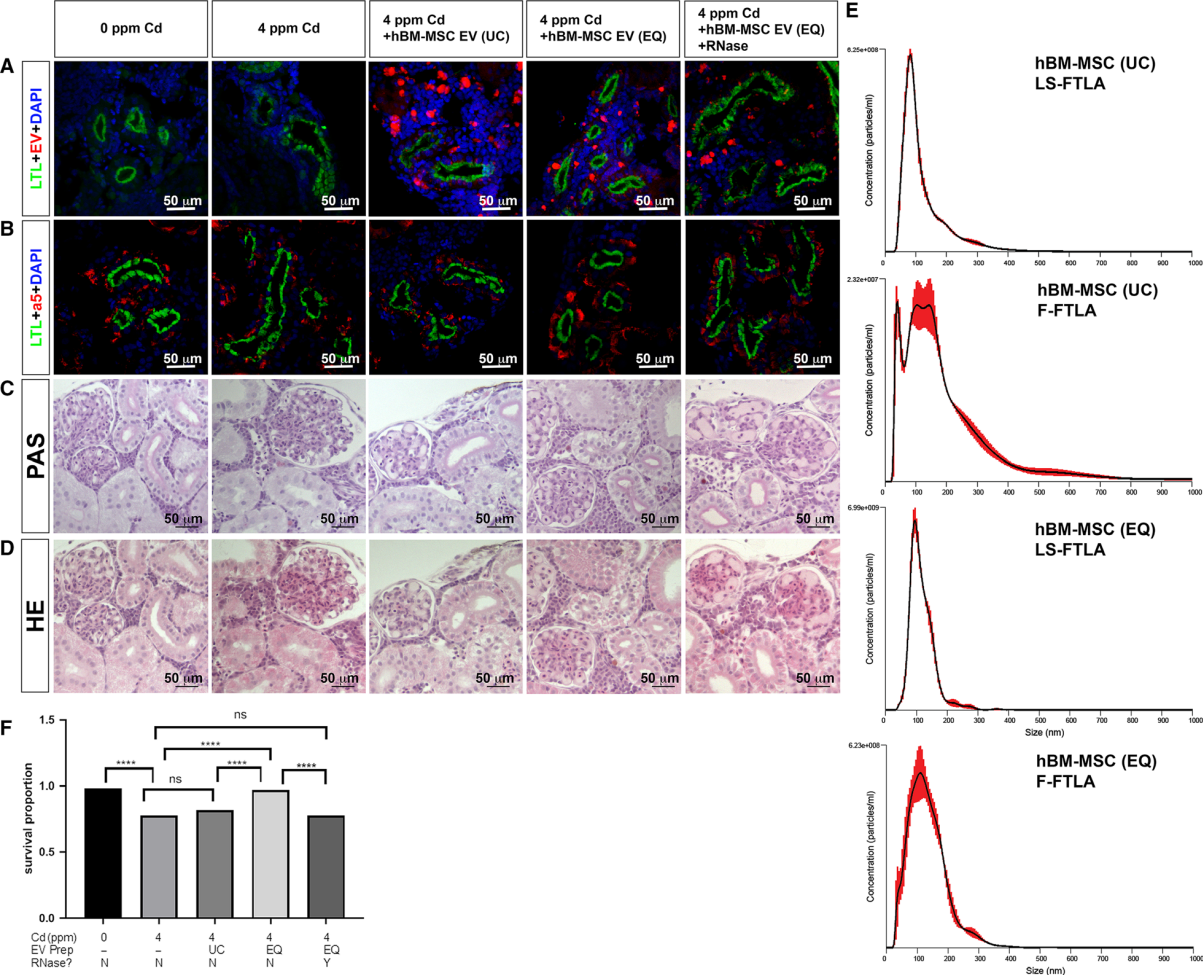
Human BM‐MSC‐derived EVs repair kidney injury in Cd‐exposed medaka. (A) LTL labeled the PT apical membrane (green). ExoGlow‐membrane red dye labeled hBM‐MSC EVs (red). DAPI was used to stain nuclei (blue). (B) LTL labeled the PT apical membrane (green). LTL (green), DAPI (blue), and a5 labeled the Na^+^, K^+^‐ATPase alpha subunit basolateral localization (red). (C) PAS staining of JB4 sections in the kidneys. (D) HE staining of JB4 sections in the kidney. (E) Fluorescence nanoparticle analysis. Finite Track Length Adjustment (FTLA), FTLA measurement for light scattering (LS‐FTLA). Regular size/concentration measurement for light scattering (LS‐SC), FTLA measurement for fluorescent data (F‐FTLA), regular size/concentration measurement for fluorescent data (F‐SC). (F) Survival proportion graph of 7 dai female medaka exposed to Cd and treated with different preparations of BM‐MSC EVs. Significance by Log‐rank test. *****P <* *0.0001; ns = not significant. The white scale bar indicates 50 μm (A, B). The black scale bar indicates 50 μm (C, D).

We evaluated the efficacy of human BM‐MSC EVs in repairing the organ damage caused by Cd exposure in our medaka model. We first compared two preparation methods of EVs that are regarded as gold standards: (1) ultracentrifugation (UC) and (2) the newly developed ExoQuick‐TC^®^ ULTRA (EQ). Both methods used 20 mL each of hBM‐MSC‐derived tissue culture medium. To measure the purity of the EVs, we compared the ratio of F‐FTLA particles/ml divided by LS‐FTLA particle/ml for both methods and found that EQ rendered a higher percentage of fluorescently labeled particles at 13.25% (1.25 × 10^11^/9.43 × 10^11^ × 100) compared with 8.94% (4.40 × 10^9^/4.92 × 10^10^ × 100) by UC (Fig. 9E). EQ purification gave a higher yield than did UC; 1.25 × 10^11^ particles and 4.92 × 10^10^ particles per batch, respectively. Additionally, Fluorescence Nanoparticle Tracking Analysis (fNTA) data showed three peaks in the UC preparation and a single peak in the EQ preparation. Thus, the EQ purification system allowed us to obtain a higher yield and a shorter processing time of 20 min compared with that of the UC method that requires several hours. From these results, we conclude that EQ results in a faster and cleaner preparation of EVs at lower costs compared to UC. Furthermore, the fNTA data indicate that the EQ purification gave less heterogeneity in the distribution of vesicle size. Overall, the EQ protocol seems superior to the classical UC method, as shown in Figure 9E.

To compare the efficacy of hBM‐MSC‐derived EVs prepared by those two methods, we injected each preparation intravenously (IV) into our medaka model at a dose of 4 × 10^7^ EV per medaka using a 2 μL injection volume after 1 day of exposure to Cd at 4 ppm. Medakas were euthanized 3 days after EV injection. To detect the EV uptake in the kidney tissue, the hBM‐MSC EVs were labeled with ExoGlow‐membrane red dye. Medaka with 4 ppm Cd exposure for 4 days without EV treatment showed uneven and wider diametric labeling of LTL on apical PT. When medakas were treated with EVs, EV uptake was detected. The tubules were almost normal with EQ‐purified EVs, whereas the tubules still evidenced abnormality with treatment with UC‐purified EVs, although this abnormality was less severe than that observed with no treatment (Fig. 9A). In contrast, RNase‐treated EVs significantly reduced the recovery of LTL‐labeled apical PT. These effects were confirmed by LTL and a5 staining in PT epithelium (Fig. 9B).

PAS (Fig. 9C) and H&E stained (Fig. 9D) JB4 histology sections showed that UC‐purified EVs repaired both PT and glomeruli abnormalities to some degree, resulting in less severe abnormalities after treatment with UC‐purified EVs. The EQ‐purified EVs treated with RNase did not repair any PT and glomeruli defects (Fig. 9). The results indicated that RNase‐sensitive molecules, such as miRNA and/or mRNA transferred from the hBM‐MSC EVs, triggered the repair process for tissue damage caused by Cd.

**Figure 10:**
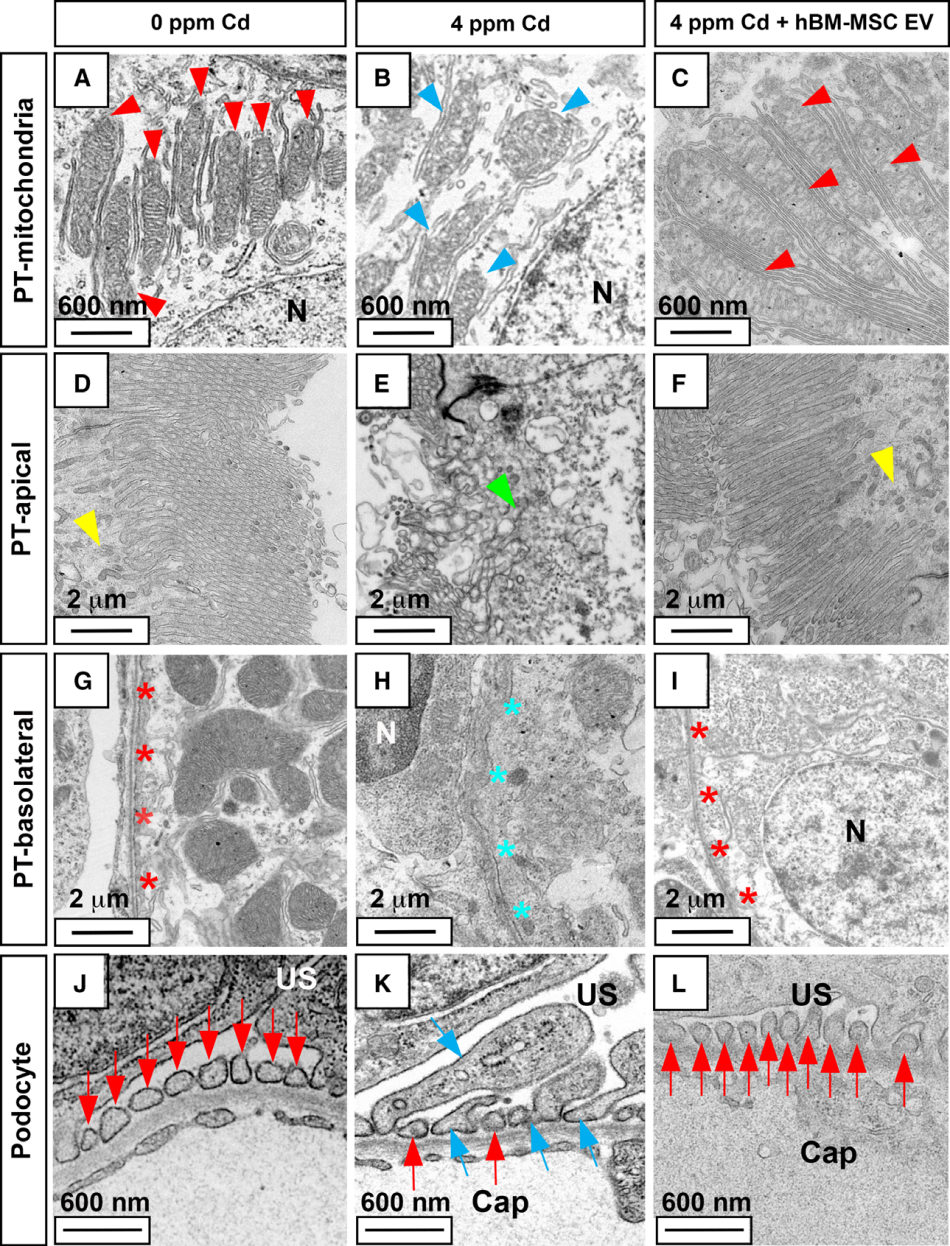
Transmission electron microscopy images for human BM‐MSC‐derived EVs repair of proximal tubule and glomerulus damage in Cd‐exposed medaka. Female 0 ppm Cd (A, D, G, J), female 4 ppm Cd (B, E, H, K), female 4 ppm Cd + human BM‐MSC‐derived exosomes (C, F, I, L). Normal proximal tubule (PT) mitochondria (red arrowhead; A, C), normal PT apical membrane vesicle (yellow arrowhead; D, F), normal PT basolateral membrane (red asterisks; G, I), normal glomeruli podocytes (red arrowhead; J, L), damaged PT mitochondria (light blue arrowhead; B), abnormal PT apical vesicles (green arrowhead; E), abnormal PT basolateral (light blue asterisk; H), enlarged podocyte foot process with apoptotic vesicles (light blue arrow; K). The scale bar indicates 600 nm (A, B, C, J, K, L) and 2 μm (D, E, F, G, H, I).

We further used TEM to examine the cellular ultrastructure to determine whether hBM‐MSC EVs purified by EQ repaired Cd exposure, altered cellular architecture, and organelles in the nephron (Ichimura et al. 2012). Significant disassembly of PT mitochondria (Fig. 10B), misshapen apical membranes (Fig. 10E), misaligned basolateral membranes (Fig. 10H), and enlarged glomerular podocytes (Fig. 10K) were observed in aged female medaka exposed to 4 ppm Cd for 4 days. As shown in Figure 6B, F, J, and N, Cd exposure suggesting pathotoxic effects on PT apical membrane, basolateral membrane, and mitochondria. IV injection of hBM‐MSC EQ‐purified EVs rescued ultrastructural damage caused by Cd (Fig. 10C, F, I and L) to an integrity level comparable to that of the control, i.e., medaka without Cd exposure on the 4th day (Fig. 10A, D, G, J). Our findings include the repair of disassembled PT mitochondria (Fig. 10B) to normal structure (Fig. 10C), misshapen and dark‐colored PT apical membranes caused by vesicle uptake (Fig. 10) repaired to normal vesicle uptake from the apical membrane (Fig. 10F), misalignment of PT epithelium (apical and basolateral membranes) (Fig. D, G) repaired to normal aligned membrane (Fig. 10F, I), and enlarged glomerular podocytes with apoptotic cells (Fig. 10K) repaired to equally distributed podocytes (Fig. 10L).

The survival rate was also improved by the treatment; the survival proportion was 0.97 for fish that received EQ‐purified EVs and 0.82 for fish that received UC‐purified EVs (Fig. 9F). The survival rate of fish that received EQ‐purified and RNase‐treated EVs dropped to 0.775, which is the same as that of no EV treatment. A similar trend of recovery in survival proportion was observed with fish that received UC‐purified EVs, from 0.775 to 0.82. When the EVs were RNase‐treated, the proportion of fish that survived decreased to 0.775 (Fig. 9F). The survival proportion of female fish exposed to 4 ppm Cd for 4 days recovered from 0.775 to 0.97 (Fig. 9F).

**Figure 11:**
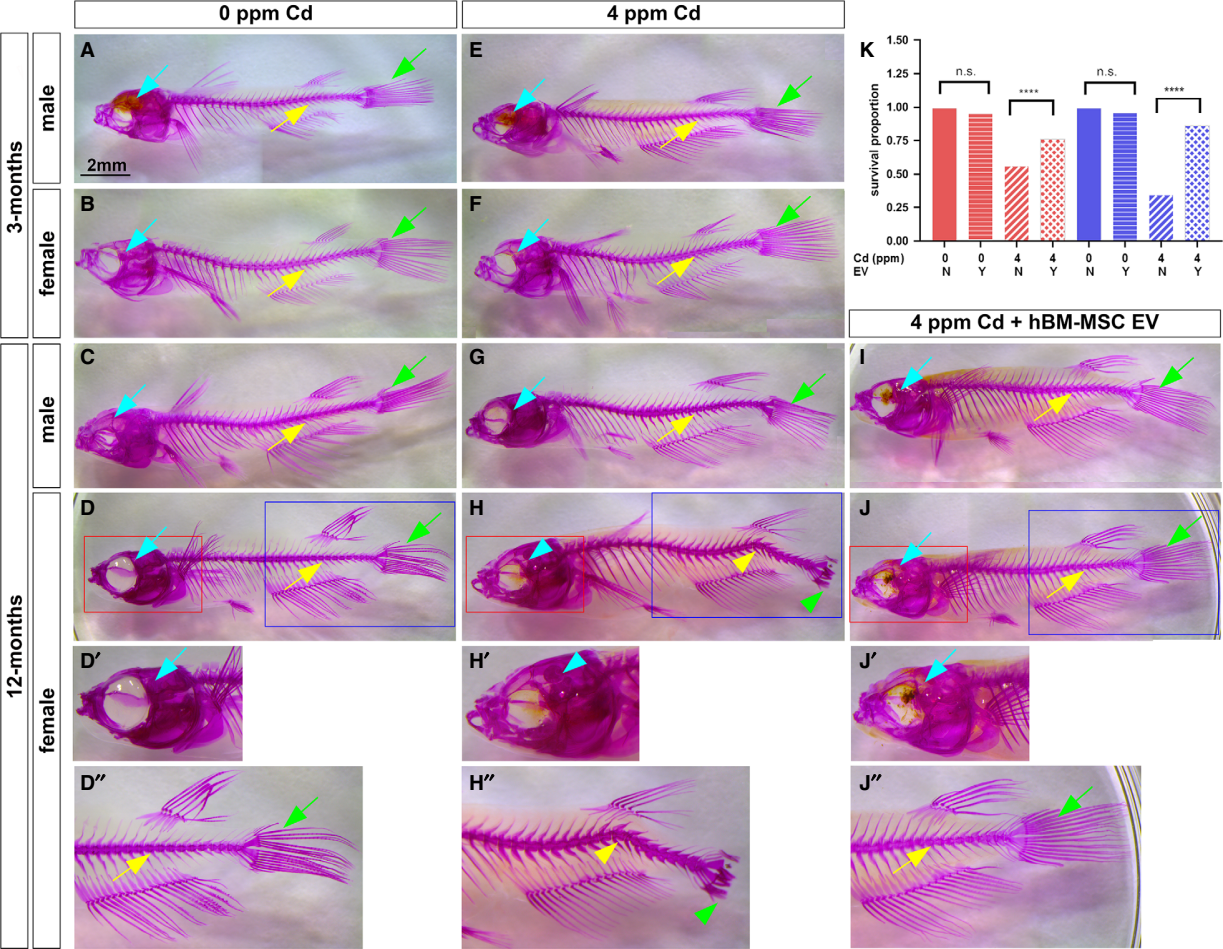
Whole‐body images of the medaka bone staining by alizarin red S. 0 ppm Cd (A, B, C, D); 4 ppm (E, F, F, G, H); 3‐month‐old medaka (A, B, E, F); 12‐month‐old medaka (C, D, G, H, I, J); human BM‐MSC‐treated (I, J); female (A, C, E, G, I); male (B, D, F, H, J); double magnified skull (red square, D’, H’, J’); double magnified vertebral column and tail fin (blue square D”, H”, J”). The scale bar for A–J indicates 2 mm. K. Survival proportion graph of 4 dai female (red) and male (blue) fish exposed to Cd and treated with extracellular vesicles (EV) or control. Normal skull (light green arrow), normal vertebral column (yellow arrow), normal tail fin (light green arrow), curved vertebral column (light blue arrowhead), short tail fin (light green arrowhead), and enlarged skull size (light green arrowhead). Survival curves were compared using Log‐rank tests. ****P <* *0.0001, ns, not significant.

We also evaluated the bone deformation caused by Cd pathotoxicity, similar to human cases reported as osteomalacia in postmenopausal women upon chronic Cd exposure (Huff et al. 2007; Lane et al. 2015; Aoshima 2016). We analyzed the whole‐mount bone staining with alizarin red S (Sakata‐Haga et al. 2018). We observed normal bone morphology at 0 ppm Cd in 3‐month‐old and 12‐month‐old female and male medaka (Fig. 11A–D, D’, D’’) and at 7 days of 4 ppm Cd exposure in 3‐month‐old female and male medaka and in 12‐month‐old male medaka (Fig. 11E–H, H’, H”). The pathotoxic effect of Cd appeared at 7 days of exposure to 4 ppm Cd in 12‐month‐old females, resulting in curved vertebral column, short tail fin and enlarged skull size (Fig. 11H, H’, H”). We evaluated the efficacy of the EV injection in repairing and/or attenuating the bone malformation. In this experiment, 12‐month‐old female and male medakas were treated with 4 ppm of Cd for 4 days and the hBM‐MSC EQ‐purified EVs were IV‐injected (Fig. 11I, J, J’, J”). Three days after injection, we examined the whole‐mount bone morphology and survival. In 12‐month‐old females, the curved vertebral column, shortened tail fin, and enlarged skull size were repaired (Fig. 11J, J’, J”), whereas the bone morphology remained normal in 12‐month‐old males (Fig. 11I), as observed at 0 ppm Cd exposure (Fig. 11C). Overall fish survival was again improved by injection with the EQ‐purified hBM‐MSC EVs (Fig. 11K). The survival proportion difference was significant (*P *<* *0.0001) between 4 ppm Cd‐exposed females (0.555) and 4 ppm Cd‐exposed females with EVs (0.76), and 4 ppm Cd‐exposed males (0.34) and 4 ppm Cd‐exposed males with EVs (0.86) (Fig. 11K). Combined, these results suggest that our EV protocol could be developed into a new therapeutic approach useful for regenerative medicine.

## Discussion

In this report, we established a system to study the pathotoxicity of heavy metal Cd using medaka of both sexes at various ages. We showed that hBM‐MSC EVs repaired kidney PT, glomerular podocytes, and bone deficiency, and significantly improved the proportion of fish that survived. Using young adult (3‐month‐old) and aged adult (12‐month‐old) medaka, we established a protocol to evaluate the organ‐damaging effect of Cd on kidney and bone, and the survival proportion at the low dose (0.5–4 ppm Cd) for a short period. Our procedure is simple, rapid, and should be useful for examining the perturbation of organ development and skeletogenesis by other xenobiotics, including different heavy metal ions such as nickel and chromium.

One advantage of our protocol is the considerably shorter time (4–7 day exposure), and low agent concentration (in our case, that of Cd) required. In pathotoxicity studies of Cd, other groups used larger animal models, such as male Sprague Dawley rats weighing 250–300 g. Due to the size of this model, a larger dose of Cd and a longer period (12 weeks) were used for daily subcutaneous injections of CdCl_2_ at a Cd dose of 0.6 mg/kg (Prozialeck et al. 2007; Edwards and Prozialeck 2009; Prozialeck et al. 2009a; Prozialeck et al. 2009b). In another case, 9‐week‐old C57BL/6NCrSlc female and male mice (body weight ~35 g) were treated with Cd‐tainted drinking water at a rate of 60 mg/kg/day for 11 weeks (Yamanobe et al. 2015). Compared with those studies using large animals, our medaka model reduces the experimental parameters substantially, requiring less Cd solution and exposure time compared with the larger animal models (Prozialeck et al. 2007; Edwards and Prozialeck 2009; Prozialeck et al. 2009a; Prozialeck et al. 2009b; Yamanobe et al. 2015; Prozialeck et al. 2016).

Using the OECD guidelines, we established an experimental system for low‐dose (0.5–4 ppm) Cd toxicity for young and aged medaka adults. The small size of medaka (3‐month‐old medaka average 0.125 g of body weight) compared with mice (8‐month‐old mice average 22 g), combined with the optimization of Cd exposure conditions, showed that medaka is an excellent model with which to observe the toxic effects of Cd on the entire body. The exposure to 4 ppm Cd for 4 days enabled us to evaluate the Cd accumulation in the blood, kidney, liver, intestine, heart, brain and genitals in medaka of both sexes. We found that the effect of Cd on liver, kidney and blood differed significantly between females and males.

Cd causes dysfunction of the kidney as a primary target. The Cd toxicity to the kidney was more evident in females than in males, as evaluated by histology on apical and basolateral membrane markers of PT, fluoro‐ruby uptake, and blood cells caused by vessel leakage. Higher doses of Cd‐induced ultrastructural deficiency in PT mitochondria and glomeruli podocytes, as revealed by TEM studies were more severe in female kidney than in male kidney. The pathotoxicity of Cd is systemic, as we confirmed that higher doses of Cd‐induced bone malformation, such as curved vertebral column, short tail fin and enlarged skull size. However, those Cd toxicities manifested only in aged (12‐month‐old) females exposed to a higher concentration of Cd (4 ppm) for 7 days.

Altogether, our results with Cd toxicity faithfully reflect the clinical case of the human malady called “*itai‐itai* disease”, to which post menopausal women are more susceptible (Aoshima 2016). This concordance strongly suggests a high clinical relevance of our medaka model for the study of age‐ and sex‐dependence of systemic Cd effects.

The increasing body of evidence indicates that EVs play a crucial role in intercellular communication in normal and pathogenic states, and probably in rejuvenating injured cells. In order to investigate the cellular signaling mechanisms of EVs, it is crucial to establish a protocol to prepare efficacious EVs and to apply them to model animals. However, the purification methods of EVs for both in vivo and ex vivo studies remain to be developed. Several methods of EV purification have been described (Riazifar et al. 2017; Castellano et al. 2017; Greening et al. 2015). Among them, the ultracentrifugation method (UC) has been considered a gold standard despite its time‐consuming protocol and the requirements of a large volume of starting materials and an expensive ultracentrifuge. Additionally, the resulting EVs tend to be contaminated with carryover proteins, such as albumins and immunoglobulins, which causes overestimation of the yield of EVs (Riazifar et al. 2017; Castellano et al. 2017; Greening et al. 2015). Seeking to establish an efficient, size‐selected EV preparation at higher yields, and to use a faster and easier method, we compared the classical UC method and the ExoQuick‐TC^®^ ULTRA (EQ) method. Our results indicate that the ExoQuick‐TC^®^ ULTRA renders a higher yield of EVs within a short period of preparation time (20 min), and the preparations had a higher content of EVs.

The highly purified human BM‐MSC EVs produced using the EQ‐method and delivered by IV injection repaired the organ damage caused by Cd toxicity, as evaluated by various morphological and histological signatures. The repair process extended overall survival significantly, which is indicative of the efficacy of EV treatment toward the systemic improvement of the diseased phenotype caused by a xenobiotic toxicity of Cd. The efficiency of EV preparation by the EQ‐method applied to our medaka model illustrates superiority toward the translational step of the protocol compared with other large animal models using rats and mice. For example, Bruno *et al.* recently showed that IV injection of UC‐purified human BM‐MSC‐derived EVs repaired kidney defects in glycerol‐induced AKI mice in 7–8 weeks during which 15 μg of EVs were injected five times (Bruno et al. 2009). Compared with the mouse model, since our protocol utilizes an adult medaka weighing about 1/176^th^ that of a mouse, the required amounts of EVs can be significantly reduced. As a result, our medaka system reduced the required EVs approximately 21.4 times, i.e., 3.5 μg EQ‐purified EVs for medaka with one IV injection versus five injections of 15 μg UC‐purified EVs for each injection, totaling 75 μg.

The simplicity and efficiency of our procedure to repair and thereby to cure damaged organs and tissues by EV injection will be useful for the study of other xenobiotics, including toxic heavy metals other than cadmium. Because of its simplicity and cost‐effectiveness, more important and complex parameters such as age association and sex dependency can easily be examined using our model, as shown here. We note that any other agents, any dietary effects causing obesity, and its associated diseases and adverse side effects of pharmaceuticals, could replace the xenobiotic agents. Also, organs other than the kidney and bone can be studied for their response to disease causing agents. Thus, our medaka model could contribute to developing a variety of new avenues for interventional strategies applicable to regenerative medicine.

## Conflict of Interest

The authors declare that there are no conflicts of interest.

## Supporting information




**Appendix S1.** ISEV guidelines for characterization of extracellular vesicles (EV).
