# Supplementary material for: Extracellular vesicles from human bone marrow mesenchymal stem cells repair organ damage caused by cadmium poisoning in a medaka model
Source: Physiol Rep. 2019 Jul 19;7(14):e14172. doi: 10.14814/phy2.14172 (PMC6642321; doi:10.14814/phy2.14172)
Supplement: Supplementary file 1 — Appendix S1. ISEV guidelines for characterization of extracellular vesicles (EV). [file PHY2-7-e14172-s001.docx]

**Supplementary Information**

**Title:**

**Extracellular vesicles from human bone marrow mesenchymal stem cells repair organ damage caused by cadmium poisoning in a medaka model**

**Authors:**

Tomomi Matsukura^1,^ ^2^, Chisako Inaba^1^, Esther A. Weygant^1^, Daiki Kitamura^2^, Ralf Janknecht^1^, Hiroyuki Matsumoto^3, 4^, Deborah P. Hyink^5^, Shosaku Kashiwada^2^ & Tomoko Obara^1*^

**Affiliations:**

^1^Department of Cell Biology, University of Oklahoma Health Sciences Center, Oklahoma City, Oklahoma, USA**,** ^2^Department of Life Sciences, Toyo University, Gunma, Japan**, ^3^**Department of Biochemistry and Molecular Biology, University of Oklahoma Health Sciences Center, Oklahoma City, OK, USA, ^4^Clinical Proteomics and Gene Therapy Laboratory, Kurume University Graduate School of Medicine, Kurume, Japan, ^5^Department of Medicine, Baylor College of Medicine, Houston, TX, USA.

**Running title:** Extracellular vesicles repair cadmium damage

***Correspondence to:**

Dr. Tomoko Obara, Department of Cell Biology, University of Oklahoma Health Sciences Center, 940 Stanton L. Young Blvd., BMSB 513A, Oklahoma City, Oklahoma 73104-5020, USA. Tel: 405-271-8001 ext. 47035, Fax: 405-271-3548, e-mail: [tomoko.obara@gmail.com](mailto:tomoko-obara@ouhsc.edu)

We have followed the ISEV guidelines for characterization of extracellular vesicles (C.Théry, K. W. Witer, et al., “Minimal Information for Studies of Extracellular Vesicles 2018 [MISEV2018]: a position statement of the International Society for Extracellular Vesicles and update of the MISEV2014 guidelines”, *J Extracell Vesicles* 2018; 7: 1535750).

**Nomenclature**

We are using “extracellular vesicle” (EV) as the generic term for particles that have been used in the manuscript, as suggested by ISEV.

**Collection and pre-processing: pre-analytical variables**

We described the respective details in the materials and methods section, as recommended by ISEV. EVs were stored at 1 μg/μl in the -80°C freezer, then diluted with phosphate buffered saline (PBS) (Fisher Scientific) prior to IV injection.

**EV separation/enrichment and concentrations**

We described the details in the materials and methods section. We used two EV separations concentrated by ultracentrifugation (UC) and ExoQuick-TC ULTRA (SBI). Enriched EVs were analyzed by Fluorescence Nanoparticle Tracking Analysis (fNTA), which is described in the materials and methods section. These data fulfill the ISEV 2018 guidelines (see their Table 1. Considerations for EV separation/enrichment).

**EV characterization: how MISEV2014 evolves in 2018**

Isolated EV quality was checked by western blot for the presence of CD63 and by NanoSight for particle size and intactness. These data fulfill ISEV 2018 guidelines (see their Table 2. Steps of EV characterization). To monitor the EV uptake into the kidney tissues, the hBM-MSCs were labeled with EV-specific dye (ExoGlow-membrane red dye [SBI]).

*New recommendation: determine the topology of EV-associated components:*

We performed RNase treatment for human BM-MSC purified by ExoQuick-TC ULTRA. The EQ-purified EVs treated with RNase did not repair any PT or glomeruli defects (Fig. 9). This result indicated that RNase-sensitive molecules, such as miRNA and/or mRNA transferred from the hBM-MSC EVs, triggered the repair process for tissue damage caused by Cd exposure in medaka. We also prepared RNA from the hBM-MSC and thereby confirmed the presence of miRNA in the EVs.

**Functional studies: how MISEV2014 evolves in 2018**

1. Dose-response studies: We prepared hBM-MSC conditioned medium before and after elimination of EVs. Only hBM-MSC conditioned medium before elimination of EVs yielded the enriched EV preparations that were also tested to repair PT and glomeruli defects in Cd-exposed medaka.
2. Negative or background controls: For a negative control, we used complete medium that had not been conditioned by cells, but was still processed in the same way as the conditioned medium. No EVs were detected.
3. Controls to assess the influence of soluble or non-EV macromolecular components: We have employed UC and EQ methods and discovered that the range of sizes of EVs were different, as determined by fNTA studies. We also labeled the hBM-MSC-derived EVs using EV-specific dye (ExoGlow-membrane red dye [SBI] and confirmed the EV uptake into the kidney tissue.
